# Impact of anti-VEGF treatment for diabetic macular oedema on progression to proliferative diabetic retinopathy: data-driven insights from a multicentre study

**DOI:** 10.1136/bmjophth-2025-002234

**Published:** 2025-07-16

**Authors:** Abraham Olvera-Barrios, Watjana Lilaonitkul, Tjebo F C Heeren, Assaf Rozenberg, Darren Thomas, Alasdair Warwick, Taha Soomro, Abdulrahman Alsaedi, Roy Schwartz, Usha Chakravarthy, Haralabos Eleftheriadis, Faruque Ghanchi, Ashish Patwardhan, Paul Taylor, Adnan Tufail, Catherine A Egan, on behalf of UK DR EHR Users group, T Akerele

**Affiliations:** 1 Medical Retina, Moorfields Eye Hospital NHS Foundation Trust, London, UK; 2 University College London Institute of Ophthalmology, London, UK; 3 University College London Global Business School for Health, London, UK; 4 Yitzhak Shamir Medical Center, Zerifin, Israel; 5 University College London Institute of Health Informatics, London, UK; 6 University College London Institute of Cardiovascular Science, London, UK; 7 Moorfields Eye Hospital NHS Foundation Trust, London, UK; 8 Ophthalmology, King Faisal Specialist Hospital and Research Centre, Riyadh, Saudi Arabia; 9 Centre for Vision and Vascular Science, Queen's University of Belfast, Belfast, UK; 10 Ophthalmology, King’s College Hospital NHS Foundation Trust, London, UK; 11 Ophthalmology, Bradford Royal Infirmary, Bradford, UK; 12 Royal Cornwall Hospitals NHS Trust, Cornwall, UK

**Keywords:** Macula, Treatment Medical, Vision, Epidemiology, Retina

## Abstract

**Objective:**

To report insights on proliferative diabetic retinopathy (PDR) risk modification with repeated antivascular endothelial growth factor (VEGF) injections for the treatment of diabetic macular oedema (DMO) in routine care.

**Methods and analysis:**

Multicentre study (27 UK-National Health Service centres) of patients with non-PDR (NPDR) and DMO. Primary outcome was PDR development. Repeated anti-VEGF injections were modelled as time-dependent covariates using Cox regression and weighted cumulative exposure (WCE) adjusting for baseline diabetic retinopathy (DR) grade, age, sex, ethnicity, type of diabetes and deprivation. PDR incidence rates (IRs) were calculated.

**Results:**

We included 2858 DMO anti-VEGF-treated eyes. Anti-VEGF injections showed a protective effect on PDR risk during the most recent 4 weeks from exposure, which rapidly decreased. Mild-NPDR had a lower PDR risk compared with moderate-NPDR (HR 1.99, 95% CI 1.13 to 3.51, p=0.015) and severe-NPDR (HR 4.63, 95% CI 2.55 to 8.41, p<0.001). Patients with type 1 diabetes showed an increased PDR risk when compared with patients with type 2 diabetes (HR 2.08, 95% CI 1.35 to 3.21, p<0.001). And every 5-year increase in age showed a 9% reduction in PDR hazards (p=0.002). The PDR cumulative IR was 4.45 (95% CI 3.89 to 5.09) per 100 person-years.

**Conclusions:**

The WCE method is a valuable modelling strategy for repeated exposures in ophthalmology. Injections are protective against PDR predominantly within the most recent 4 weeks. Based on observed data, we show that age and baseline DR severity are relevant predictors of poor outcomes in patients with DMO treated with anti-VEGF.

**WHAT IS ALREADY KNOWN ON THIS TOPIC**

**WHAT THIS STUDY ADDS**

- The impact of anti-VEGF on PDR risk varies based on the timing of exposure, and the effect is not permanent.
- Despite repeated treatments with anti-VEGF injections, patients with DMO may still progress to PDR.

**HOW THIS STUDY MIGHT AFFECT RESEARCH, PRACTICE OR POLICY**

- Our work underscores the significance of taking into account repeated treatments at varying time intervals in ophthalmology, highlighting the utility of the weighted cumulative exposure method.
- Implementing adequate modelling strategies to address the complexities of exposures in clinical settings can improve predictions and patient outcomes.
- We provide PDR incidence rates of importance for power calculations and future research.

## Introduction

 Proliferative diabetic retinopathy (PDR) and diabetic macular oedema (DMO) are among the leading causes of incident sight impairment and blindness in the working age population.^1 2^

Treatment of DMO and PDR has evolved over the last few decades after pivotal laser treatment trials.^3 4^ Anti-vascular endothelial growth factor (VEGF) therapy has become the standard of care for managing DMO.^5^ Notably, DMO can be present in cases with any degree of diabetic retinopathy (DR), and evidence has shown that anti-VEGF injections reduce DR progression rates and improve DR severity score (DRSS).^4^,^8^ However, despite rigorous and intensive intravitreal anti-VEGF DMO treatment protocols in clinical trials, progression to PDR still occurs in treated eyes, though at lower rates.^4 5 8^ Moreover, preventive treatment with anti-VEGF in patients with non-PDR (NPDR) and no DMO shows that DMO and/or PDR incidence risk is not eliminated.^4^,^7^

Little is known about how DMO anti-VEGF treatment regimens impact PDR risk outside clinical trials, whether the effect of repeated anti-VEGF injections accumulates or persists after treatment cessation, and whether the same PDR surveillance recommendations apply for DMO treated eyes. In this context, accounting for the exposure to anti-VEGF injections is important in patients with DR. Estimating the effect of intravitreal anti-VEGF for DMO treatment on PDR risk with real-world data is challenging due to confounding, variability in treatment regimens, uncertainty about cumulative effect, and the uncertain relevance of injections given at different time intervals. Methods that incorporate duration, number and timing of exposures have been suggested as the preferred approach to account for exposure effects.^9 10^ Novel methods model the history of drug exposure, such as the weighted cumulative exposure (WCE), flexibly representing the exposure’s past effect on current risk through recency-weighted exposures.

The present study addresses a significant gap in our knowledge by utilising real-world, rather than modelled, data to examine the impact of anti-VEGF exposures on progression to PDR in individuals where the indication for anti-VEGF injections was DMO. We report PDR incidence rates (IRs) at the point of care to support clinical decision-making for patients receiving courses of intravitreal injections, which may last for years, and may occur in treatment pathways where the peripheral retina is not examined at every clinic visit.

## Methods

### Study design and setting

This was a retrospective multicentre study. The UK DR Electronic Health Record (EHR) User Group gathers large-scale routine clinical data to improve the prevention, diagnosis and treatment of DR. 27 UK centres using the same EHR system (Medisoft, Leeds UK), with mandated structured DR feature grading and time-stamped interventions (eg, intravitreal injections, laser procedures and ophthalmic surgeries), contributed data. An algorithm generates DR grades according to Early Treatment Diabetic Retinopathy Study (ETDRS),^11^ International^12^ and English National Screening Committee (UK)^13^ DR classification systems. Each site is the only English National Health Service provider of DR care for the local population and very few patients switch between providers or access private care, particularly when there is need for ongoing treatment.^14^ All patients were new referrals from the English diabetic eye screening programme (DESP), a nationwide systematic programme maintained by rigorous quality assurance measures.

### Data extraction

Anonymised data were remotely extracted through the EHR’s DR module from the time of their first DR structured assessment entry onto the EHR, as described by Egan *et al*,^15^ to the date of their last clinical entry before the data extraction on 31 December 2018.^15^ Demographic data were extracted from the hospital’s patient administration system to the EHR. The cohort went through a staged exclusion process (online supplemental figure 1). The primary analysis included eyes with NPDR and DMO treated with anti-VEGF. Secondary analyses additionally included NPDR treatment-naïve eyes without DMO. Eyes with PDR at baseline or at time of first injection were excluded. Eyes with DMO treated with anti-VEGF which did not complete a loading dose of at least three injections and eyes with indications for anti-VEGF treatment other than DMO were excluded.

The recording of clinical variables^16^ included age, sex, type of diabetes (categorised as type 1, type 2 and other), ethnicity (categorised as white, black, any other Asian, other and not stated) and deprivation, measured as the index of multiple deprivation (IMD), the official measure of relative deprivation in England.^16 17^ Composite ETDRS scores were automatically generated in the EHR. For analysis, DR severity in each eye was graded at clinic visits as mild-NPDR (ETDRS levels 20–35), moderate-NPDR (ETDRS level 43), severe-NPDR (ETDRS levels 47–53) and PDR (ETDRS levels 61–81).

### Statistical analysis

The main outcome measure was PDR development, defined as retinal or optic nerve new vessels (NV), NV of the iris or angle, neovascular glaucoma, tractional retinal detachment, vitreous haemorrhage, preretinal haemorrhage or record of pan-retinal photocoagulation or vitrectomy for PDR-related reasons. The primary analysis assesses the effect of past intravitreal injections on PDR risk (n=2858). As secondary analyses, we report PDR IR in DMO anti-VEGF-treated eyes (n=2858) and in a contemporary treatment-naïve NPDR cohort without DMO (n=50 376).

One eye per patient was included for the primary analysis as follows: (1) the first anti-VEGF-treated eye; (2) if the first anti-VEGF treatment was bilateral, the eye with worse DR grade was selected and (3) if the first treatment was bilateral and the DR grade was the same bilaterally, an eye was selected at random. In treatment-naïve patients without DMO, eyes were identified as follows: (1) the eye with worse DR grade was included; (2) and if the DR grade was the same bilaterally, a random selection was carried out. Eyes were censored on change in their treatment modality (other than anti-VEGF in eyes with DMO), intraocular surgery or their last DR assessment/follow-up.

### Effect of past anti-VEGF exposures on risk of PDR

To examine the impact on model fit when accounting for repeated exposures in treated eyes, we implemented three different modelling strategies with different degrees of complexity using Cox regression and the novel WCE method in anti-VEGF-treated DMO eyes (n=2858).^18^ The proportionality assumption was tested graphically by inspection of Schoenfeld residuals.^19^ All models adjusted for baseline DR severity, age, sex, ethnicity, type of diabetes and IMD. We compared goodness of fit using Akaike information criterion (AIC) while accounting for differences in degrees of freedom.^18 20^ Lower AIC values indicate a better fit and differences in AIC above 10 units are considered significant.^20^ Briefly, a simpler model with higher AIC (worse fit) would likely suffer from under-fit bias when compared with a more complex model with a lower AIC (better fit).^21^

A traditional Cox_model_ adjusted for all time-fixed covariates and ignored anti-VEGF exposures. Cox_model_ allowed comparisons with models adjusting for anti-VEGF injections. Using an extension of the Cox model (Cox_tdc_), anti-VEGF treatments were modelled as a time-dependent unweighted cumulative sum of exposures.^19 22^

In pharmacoepidemiology, current risk may be affected by a recent increase in exposures.^23^ Due to uncertainty about how past anti-VEGF injections impact PDR risk, we used weight functions fitted to the data using flexible restricted cubic regression splines modelled in Cox regression, avoiding a priori assumptions about the specific shape of the weight function following the methods from Sylvestre and Abrahamowicz^18^ (WCE_model_). We considered five different windows of aetiologically relevant exposure. For each time window, alternative models, with 1–3 interior knots uniformly placed across the time window were compared. The length of time window before index date and number of internal knots were chosen from the model with best fit assessed by AIC (where minimum AIC means a better fit).^18 20 21^ The linear combination of estimates from the weight function was used to calculate a WCE score for each individual (online supplemental methods). The WCE_model_ controlled for time-fixed covariates and for each patient’s WCE score.^18^

### IRs of PDR in anti-VEGF-treated eyes with DMO versus treatment-naïve eyes without DMO

In addition to DMO presence, inherent differences between anti-VEGF-treated DMO eyes and treatment-näive eyes without DMO were expected a priori. Baseline age, DR severity grade and type of diabetes showed standardised mean differences greater than 0.1, suggesting imbalances (online supplemental table 1). Younger individuals with greater moderate to severe-NPDR and type 2 diabetes were more frequently seen in the DMO patients. Ethnicity also showed imbalances with ‘not stated’ ethnicity proportion being smaller in eyes with DMO.

Henceforth, cumulative (table 3) and period (online supplemental table 2) IR by DMO status and identified risk factors were calculated to illustrate PDR transition probabilities at the point of care.

All analyses were performed using R V.4.0.3. The survival^24^ and WCE^18^ packages were used for survival analyses.

## Results

A total of 2858 eyes from 2858 patients (1743/2858, 61% male) with NPDR and anti-VEGF-treated DMO were included in the primary analysis. A total of 50 376 treatment-naïve eyes without DMO from 50 376 patients (26 996/50 376, 54% male) were included as part of secondary IR analyses (online supplemental figure 1). Table 1 shows cohort characteristics; 50% (26 823/53 234) of patients self-described as white, and almost one-third (31%, 16 545/53 234) were in the most deprived quintile of deprivation (first quintile). Overall, there were 7992 incident PDR cases over a median (IQR) follow-up of 2.3 (1.1–4.4) years.

**Table 1:** Cohort characteristics

Characteristic	OverallN=53 234	Group	
		DMO anti-VEGF-treatedN=2858	Treatment-naïve without DMON=50 376
Age	61 (51, 71)	64 (57, 72)	61 (51, 71)
Sex			
Female	22 145 (42%)	1115 (39%)	21 030 (42%)
Male	31 089 (58%)	1743 (61%)	29 346 (58%)
Baseline DR grade			
Mild NPDR	19 895 (37%)	463 (16%)	19 432 (39%)
Moderate NPDR	28 931 (54%)	1935 (68%)	26 996 (54%)
Severe NPDR	4408 (8.3%)	460 (16%)	3948 (7.8%)
Ethnicity			
Any other Asian	1281 (2.4%)	61 (2.1%)	1220 (2.4%)
Black	3673 (6.9%)	209 (7.3%)	3464 (6.9%)
Not stated	14 432 (27%)	544 (19%)	13 888 (28%)
Other	951 (1.8%)	71 (2.5%)	880 (1.7%)
South Asian	6074 (11%)	313 (11%)	5761 (11%)
White	26 823 (50%)	1660 (58%)	25 163 (50%)
Type of diabetes			
Type 2	34 095 (64%)	2129 (74%)	31 966 (63%)
Type 1	7078 (13%)	183 (6.4%)	6895 (14%)
Other	1757 (3.3%)	66 (2.3%)	1691 (3.4%)
Unknown	10 304 (19%)	480 (17%)	9824 (20%)
IMD (quintiles)			
1	16 545 (31%)	915 (32%)	15 630 (31%)
2	12 906 (24%)	688 (24%)	12 218 (24%)
3	9121 (17%)	479 (17%)	8642 (17%)
4	7804 (15%)	383 (13%)	7421 (15%)
5	6858 (13%)	393 (14%)	6465 (13%)

Median (IQR) for continuous variables.

Count (column %) for categorical variables.

DMO, diabetic macular oedema; DR, diabetic retinopathy; IMD, Index of Multiple Deprivation; NPDR, non-proliferative diabetic retinopathy; VEGF, vascular endothelial growth factor.

There were 209 incident PDR cases over a median (IQR) follow-up of 1.2 (0.6–2.4) years in DMO anti-VEGF-treated eyes. The median (IQR) number of intravitreal injections was 11 (7–19); 11 (6–18) for mild-NPDR eyes, 12 (7–18) for moderate-NPDR and 12 (7–23) for severe-NPDR. The median number of injections by baseline DR severity per year of follow-up is shown in online supplemental table 3. Most patients received ranibizumab (67%, 1921/2858) at baseline, followed by aflibercept (31%, 889/2858), and bevacizumab (2%, 48/2858).

In treatment-naïve eyes without DMO, there were 7783 incident PDR cases over a median (IQR) follow-up of 2.4 (1.1–4.5) years.

### Effect of past anti-VEGF exposures

The WCE_model_ with a regression spline function based on three equally spaced knots over a 6-month time window exposure showed the best fit to the data (AIC=2837.6, online supplemental tables 4 and 5) when compared with Cox_model_ and Cox_tdc_. Online supplemental figure 2 shows the estimated weight function for the WCE_model_, which illustrates the relative strength of anti-VEGF impact on PDR risk. Current and most recent doses showed the highest protective effect (negative weights). This effect rapidly decreases with increasing time after exposure, reaching 0 at approximately week 4. More remote doses did not seem to have an impact on current risk of PDR. We report mutually adjusted HR from the WCE_model_ (table 2). The poorest fit to the data was seen in Cox_model_ which ignored the exposure of intravitreal injections (AIC difference of 94 vs WCE_model_). Mutually adjusted HRs from Cox_model_ and Cox_tdc_ are provided in online supplemental table 6.

**Table 2:** Mutually adjusted hazard ratios allowing for baseline diabetic retinopathy grade, age, sex, type of diabetes, ethnicity and index of multiple deprivation.

Characteristic	HR (95% CI)	P value
Baseline DR grade		
Mild NPDR	1.00	
Moderate NPDR	1.99 (1.13 to 3.51)	**0.015**
Severe NPDR	4.63 (2.55 to 8.41)	**2.8e–07**
Age (per 5-year increase)	0.91 (0.85 to 0.96)	**0.002**
Sex		
Female	1.00	
Male	1.36 (1.00 to 1.84)	**0.047**
Type of diabetes		
Type 2	1.00	
Type 1	2.08 (1.35 to 3.21)	**7.3e–04**
Other	1.24 (0.50 to 3.10)	0.639
Unknown	0.89 (0.60 to 1.32)	0.552
Ethnicity		
White	1.00	
South Asian	0.91 (0.58 to 1.45)	0.698
Black	0.44 (0.20 to 0.96)	**0.036**
Any Other Asian	0.80 (0.29 to 2.21)	0.657
Other	0.34 (0.08 to 1.42)	0.132
Not stated	1.02 (0.70 to 1.48)	0.917
IMD quintiles		
1 (most deprived)	1.00	
2	1.03 (0.72 to 1.49)	0.867
3	1.03 (0.68 to 1.56)	0.903
4	0.87 (0.56 to 1.36)	0.542
5 (least deprived)	0.62 (0.37 to 1.04)	0.064

Anti-VEGF exposures are modelled as cumulative exposures weighted by recency.

Bold p-values showed statistically significant results.

CI, Confidence interval; DR, diabetic retinopathy; HR, Hazard ratio; IMD, Index of Multiple Deprivation; NPDR, non-proliferative diabetic retinopathy; VEGF, vascular endothelial growth factor.

Eyes with severe-NPDR at baseline showed a more than 4-fold increase in PDR hazards (HR 4.63, 95% CI 2.55 to 8.41, p=2.8×10^–7^) when compared with mild-NPDR patients (table 2). Every 5-year increase in age showed a 9% reduction in hazards of PDR (95% CI 0.85 to 0.96; p=0.002). PDR hazards in patients with type 1 diabetes were 2.08-fold higher when compared with type 2 diabetes patients (p=7.3×10^–4^). Compared with females, males showed a 36% increase in PDR hazards (p=0.047). Black patients showed a 56% reduction in PDR hazards when compared with white participants.

A subanalysis evaluated DR features (dot-blot haemorrhages in 4 quadrants (reference), venous beading, and intraretinal abnormalities (IRMA)) associated with PDR progression in anti-VEGF-treated eyes with severe-NPDR and DMO (460/2858, 16%). IRMA showed a significant association with PDR (HR 2.55, 95% CI 1.12 to 5.79, p=0.022) when compared with dot-blot haemorrhages in 4 quadrants (online supplemental table 7). However, IRMAs were present in 71.2% (325/460) of eyes. Venous beading did not show a significant association with PDR.

### Progression to PDR

At the first recorded visit, the point prevalence of overall PDR was 1.7% (1846/106 491, online supplemental figure 1). The cumulative IR (CIR) of PDR in NPDR anti-VEGF-treated DMO eyes was 4.45 (95% CI 3.89 to 5.09) per 100 person-years). The CIR of PDR in NPDR treatment-naïve eyes without DMO was 5.13 (95% CI 5.13 to 5.13) per 100 person-years). Table 3 and online supplemental table 2 show CIRs and period IRs of PDR per 100 person-years by follow-up in NPDR anti-VEGF-treated DMO eyes and NPDR treatment-naïve eyes without DMO, respectively. Baseline DR severity showed a strong relationship with PDR. Progression to PDR in yearly intervals showed a monotonic increase overall. Younger individuals consistently showed higher PDR rates when compared with older patients.

**Table 3:** Cumulative incidence rates per 100 person-years for proliferative DR development by length of follow-up with 95% CIs

Characteristic	Year 1		Year 2		Year 3		Year 4	
	Non-DMO*	DMO†	Non-DMO*	DMO†	Non-DMO*	DMO†	Non-DMO*	DMO†
Overall	3.09 (2.96 to 3.21)	4.09 (3.48 to 4.70)	3.94 (3.80 to 4.08)	4.64 (4.00 to 5.29)	4.47 (4.32 to 4.62)	4.48 (3.84 to 5.11)	4.73 (4.57 to 4.88)	4.57 (3.93 to 5.22)
Age group								
<55	4.12 (3.87 to 4.37)	6.82 (5.02 to 8.61)	5.09 (4.81 to 5.37)	7.99 (6.06 to 9.93)	5.77 (5.47 to 6.06)	7.86 (5.94 to 9.78)	6.10 (5.80 to 6.40)	8.03 (6.09 to 9.96)
55 to <65	3.24 (2.98 to 3.50)	3.35 (2.37 to 4.33)	4.11 (3.82 to 4.40)	4.38 (3.27 to 5.50)	4.56 (4.25 to 4.86)	4.19 (3.10 to 5.28)	4.81 (4.50 to 5.12)	4.06 (2.99 to 5.14)
≥65	2.11 (1.95 to 2.28)	3.52 (2.71 to 4.32)	2.83 (2.64 to 3.02)	3.48 (2.68 to 4.28)	3.25 (3.05 to 3.46)	3.29 (2.51 to 4.07)	3.41 (3.20 to 3.62)	3.49 (2.69 to 4.30)
Sex								
Female	3.11 (2.91 to 3.30)	3.36 (2.47 to 4.25)	3.83 (3.61 to 4.05)	4.02 (3.06 to 4.99)	4.28 (4.05 to 4.51)	3.67 (2.74 to 4.60)	4.53 (4.29 to 4.76)	3.69 (2.76 to 4.62)
Male	3.07 (2.91 to 3.24)	4.57 (3.75 to 5.39)	4.02 (3.83 to 4.21)	5.06 (4.19 to 5.92)	4.61 (4.41 to 4.81)	5.01 (4.15 to 5.87)	4.87 (4.66 to 5.08)	5.16 (4.29 to 6.03)
Baseline DR grade								
Mild NPDR	1.17 (1.04 to 1.30)	2.13 (1.02 to 3.23)	1.54 (1.40 to 1.69)	2.05 (0.97 to 3.14)	1.87 (1.71 to 2.03)	1.72 (0.73 to 2.71)	2.13 (1.96 to 2.30)	1.86 (0.83 to 2.89)
Moderate NPDR	3.34 (3.16 to 3.52)	3.24 (2.58 to 3.90)	4.46 (4.25 to 4.67)	3.98 (3.25 to 4.71)	5.05 (4.83 to 5.27)	3.91 (3.18 to 4.63)	5.32 (5.10 to 5.55)	4.06 (3.32 to 4.80)
Severe NPDR	10.88 (10.06 to 11.69)	9.65 (7.39 to 11.92)	12.27 (11.41 to 13.13)	10.25 (7.92 to 12.57)	13.48 (12.59 to 14.37)	9.86 (7.57 to 12.15)	13.64 (12.74 to 14.54)	9.64 (7.38 to 11.91)

* Shows cumulative incidence rates for patients with no DMO and no anti-VEGF injections.

† Shows cumulative incidence rates for patients with DMO treated at the point of care with anti-VEGF injections.

DMO, diabetic macular oedema; DR, diabetic retinopathy; NPDR, non-proliferative diabetic retinopathy; VEGF, vascular endothelial growth factor.

**Figure 1:**
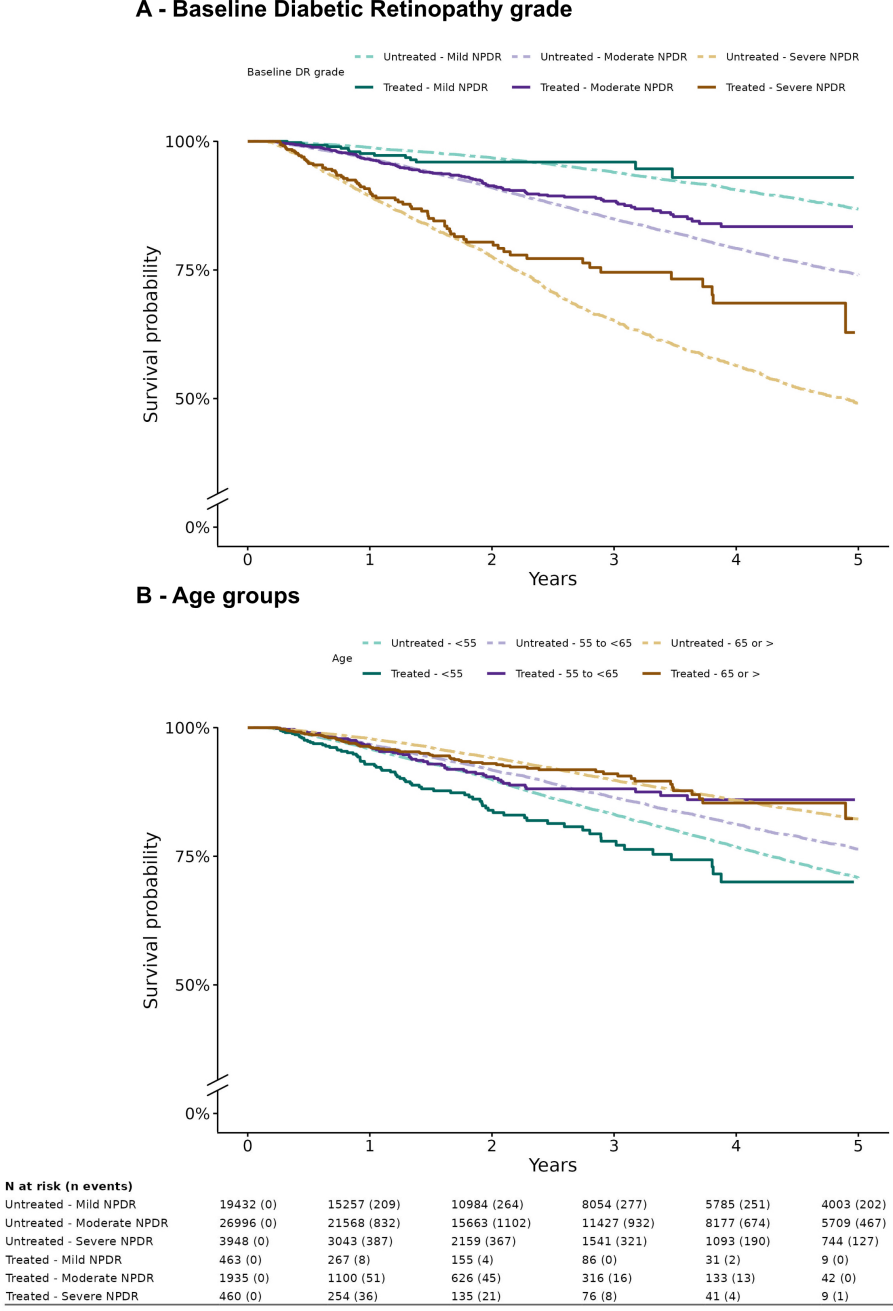
Survival curves for progression to proliferative diabetic retinopathy (PDR) stratified by anti-VEGF treatment status (‘Treated’: anti-VEGF-treated DMO eyes; ‘Untreated’: treatment-naïve eyes without DMO) and baseline non-PDR (NPDR) severity (**A**), and age (**B**). DMO, diabetic macular oedema; VEGF, vascular endothelial growth factor.

Figure 1A shows PDR development probabilities stratified by baseline DR severity. By year 3, 4% of eyes with DMO and mild NPDR, and 6% of eyes with mild NPDR without DMO progressed to PDR compared with 25% of eyes with DMO and severe NPDR, and 35% of eyes with severe NPDR without DMO. Figure 1B shows PDR development probabilities stratified by age categories. By year 3, 9% of eyes with DMO of patients ≥65 years, and 10% of eyes of patients more than ≥65 years without DMO progressed to PDR, compared with 22% of eyes with DMO of patients <55 years, and 17% of eyes of patients <55 years without DMO.

## Discussion

This study provides a comprehensive analysis of PDR development in eyes with DMO undergoing anti-VEGF treatment in routine clinical practice. Our findings support the use of WCE to model the complex time-dependent exposures to anti-VEGF injections, preventing underfitting in real-world data. Our research sheds light on the duration of the protective effect of anti-VEGF injections, particularly during the most recent 4 weeks from treatment (online supplemental figure 2). Importantly, despite frequent intravitreal injections, DMO eyes treated with anti-VEGF still develop PDR at similar transition rates when compared with eyes without DMO (table 3). Our findings underscore the need for peripheral retinal examination, regardless of repeated anti-VEGF injections, for new onset neovascularisation following the established recommended screening intervals based on DR severity: likely to be important for ‘injection-only’ clinical pathways.

### The effect of intravitreal anti-VEGF injections on PDR progression

Incorporating intravitreal anti-VEGF exposures as weighted time-dependent covariates resulted in improved goodness of fit to the data (online supplemental table 5), providing evidence that PDR risk is modified by anti-VEGF exposures and varied with treatment recency. The RISE (Ranibizumab injection in subjects with clinically significant macular edema with center involvement secondary to diabetes mellitus) and RIDE (Ranibizumab injection in subjects with clinically significant macular edema with center involvement secondary to diabetes mellitus) extension study^8^ demonstrated that patients with DRSS stability/improvement received more injections compared with those with worsened scores (p<0.001). Furthermore, greater instability occurred in more severe baseline cases (p<0.001).^8^ The PANORAMA (Study of the Efficacy and Safety of Intravitreal Aflibercept for the Improvement of Moderately Severe to Severe Nonproliferative Diabetic Retinopathy) study suggested a dose-dependent effect on DRSS improvement,^7^ supporting the importance of past anti-VEGF exposures and baseline DR severity. We show that the protective anti-VEGF effect declines sharply, becoming insignificant at approximately 4 weeks after exposure. Reduction in treatment frequency to less-than-monthly injections, or treatment discontinuation, translates to an increase in PDR risk compared with eyes treated with continued monthly injections. Special attention would be warranted for high-risk groups stopping treatment.

### Progression to PDR

Nguyen *et al*^25^ simulated treating severe-NPDR with rigorous early anti-VEGF injections versus delaying treatment until PDR development using extrapolated 1-year data from PANORAMA^7^ and RISE/RIDE^26^ trials. Based on real-world data from over 77 000 patients, a 2 million NPDR patient cohort was simulated. Early treatment showed a 51.7% relative risk reduction in PDR development over 5 years and a 19.4% absolute risk reduction. The authors highlight the lack of real-world evidence of anti-VEGF treatment on risk of PDR. However, as only 1-year RCT data are extrapolated in a Monte Carlo model, no differences in the timing and frequency of the treatments were taken into account, and results are likely to differ in real-world settings where intensity and timing of treatment vary. We address this by providing point-of-care IR of progression to PDR in both, eyes with NPDR and DMO treated with anti-VEGF and in treatment-naïve eyes with NPDR and without DMO, and by providing real-world pharmacoepidemiological evidence of the importance of past anti-VEGF treatments (table 3, online supplemental figure 2).

The PANORAMA study^7^ investigated the effect of longer anti-VEGF treatment intervals for patients without DMO. The results showed a 2-year cumulative probability of PDR of 13.5% in treated eyes, compared to 33.2% in sham eyes. In the RISE/RIDE studies,^4^ 8% (21/257) of cases in the sham group progressed to PDR at 2 years follow-up from NPDR at baseline, compared with 2% (10/502) of those treated with ranibizumab. Total progression of retinopathy in RISE/RIDE by all measures was 30% (78/257) in the sham group and 10% (51/502) in the treated groups. The DRCRnet protocol W^6^ explored the potential use of anti-VEGF injections at earlier stages of retinopathy to prevent DR progression in eyes without DMO, finding 2-year PDR cumulative probability of 13.5% in the aflibercept treated group. The PDR cumulative probability at year 2 in our study was 7.9% in the treatment-naïve eyes without DMO (CIR 5.13, 95% CI 5.13 to 5.13 per 100 person-years) and 9.7% in the anti-VEGF-treated DMO eyes (CIR 4.45, 95% CI 4.44 to 4.46 per 100 person-years). Possible reasons for the higher PDR cumulative probabilities in anti-VEGF-treated DMO eyes in our study at year 2 are the differences in baseline characteristics (with younger individuals and higher proportion of moderate to severe NPDR in patients with DMO, online supplemental table 1) and the variation in appointment and treatment intervals in routine care. Unlike clinical trials, we provide cumulative and period PDR risk estimates for up to 4 years of follow-up.

In addition to identifying DR severity as a predictor of PDR outcomes, it is important to note that younger patients with DMO showed faster progression rates to PDR during the first 3 years of follow-up, despite anti-VEGF treatment, when compared with patients without DMO (figure 1B, table 3). Based on the observed CIRs (table 3) and insights on the importance of past intravitreal anti-VEGF exposures on risk of PDR (online supplemental figure 2), review under mydriasis for NPDR eyes with DMO undergoing anti-VEGF treatment should not be delayed more than the established recommendations of point of care DR screening (ie, every 12 months for mild-NPDR, 6 months for moderate-NPDR and 3–4 months for severe-NPDR) and account for age of the participant on a case-by-case basis. Although in the long term, novel grading systems incorporating standard^27^ and widefield optical coherence tomography angiography may be resilient to the effect of anti-VEGF in their predictive power and may eventually supplant the ETDRS grading system, it will take many years to curate datasets to validate such systems. Currently, in the absence of novel grading systems, this study sheds light on PDR development rates in anti-VEGF-treated DMO eyes and treatment-naïve eyes without DMO, enriching the evidence for surveillance needs given the prevalence and variation in dosing and intervals of injection treatments for DMO at the point of care.^28^

### PDR associations

Baseline DR severity is one of the most relevant biomarkers for predicting patient outcomes, even with frequent anti-VEGF injections. Baseline DR severity grade was associated with 1.99-fold and 4.63-fold rises in PDR hazards for moderate and severe-NPDR when compared with mild-NPDR, respectively. IRMA showed a 2.55-fold risk of progressing to PDR when compared with dot-blot haemorrhages in 4 quadrants, further highlighting the relevance of clinical DR features.^11 16 29^

Younger patients were at increased risk for developing PDR, confirming the findings of other studies.^30 31^ Males showed a 36% increase in PDR hazards when compared with females. Previous studies on DR and DMO have not shown significant sex differences. Sex is a known confounder in diseases involving the microvasculature,^16 32^ and together with evidence of a reduced likelihood of achieving metabolic therapeutic goals,^30^ are possible reasons for the observed differences. Black patients have been reported to be more likely to develop DR than white patients, more likely to present with sight-threatening DR,^33 34^ and less likely to attend for diabetic eye screening.^35^ Interestingly, white patients showed worse survival when compared with patients of black ethnicity, however, 19% (544/2858) of patients had missing ethnicity data, and ethnic associations should be interpreted with caution. Type 1 diabetes and worse DR severity at baseline were the strongest predictors of PDR development.

### Strengths and limitations

This study has several strengths. First, we examined a large cohort with structured data collection in EHR including retinopathy grade, retinal features, time-stamped diagnoses, clinical examinations and treatments/procedures. Second, we have analysed the effect of intravitreal injections given in routine clinical care settings rather than modelled data on PDR development by modelling injections as weighted exposures in Cox regression using the novel WCE method. Third, we provide IRs of development of PDR in both, anti-VEGF-treated DMO eyes and treatment-naïve eyes without DMO, by relevant characteristics, which are also of significance for power calculations for clinical trials.

The main limitations of our study are as follows. Our data are dependent on the quality of EHR examination and recordings. The validity of our findings is therefore subject to the quality and completeness of the clinical information documented during routine care. This includes the potential for measurement inaccuracies, coding errors, and missing data, which are inherent to retrospective EHR-based research. While the large scale of our dataset may mitigate the impact of random errors, it cannot eliminate potential systematic biases within the clinical record. A second limitation of our study is that control of diabetes could have improved in the most recent years, and different drugs have come into play since the date of data extraction (31 December 2018), however, we only included cases which had completed the loading anti-VEGF course, and with a median (IQR) of 6 (4–7) injections during the first year, the treatment patterns in the current cohort fall in line with an independent large study using real-world data.^36^ A third limitation is that comparing the progression rates of eyes with DMO that are untreated versus eyes with DMO treated with anti-VEGF is inherently challenging in a clinical setting where anti-VEGF is the mainstay of treatment for clinically significant DMO. However, our point-of-care CIR and period IR estimates provide a valuable contribution to the literature and stress the need for continued DR severity grading under mydriasis in anti-VEGF-treated patients. A fourth limitation is the proportion of usable ethnicity records (73%) which warrants cautious interpretation of the evidenced associations between ethnicity and PDR. Fifth, we did not conduct a subanalysis stratifying PDR risk by specific anti-VEGF agent due to the real-world nature of our data and rates of treatment switching. Switching occurred in 77.1% (11/48) of eyes starting Bevacizumab, 34.8% (1253/1921) starting Ranibizumab, and 5.5% (840/889) starting Aflibercept. We omitted this stratification to avoid introducing bias associated with time-varying exposures, changes in prescribing patterns, and confounding by indication. Lastly, data on confounders such as glycaemic control, blood pressure readings and other systemic factors which are independently associated with PDR development were not collected as part of this large database. As with any observational study, results can be influenced by residual unaccounted confounders that can only be resolved by a randomised controlled trial of differing intensity and duration of treatment regimens for approved anti-VEGF agents.

## Conclusions

Our results support the use of WCE in modelling the effects of anti-VEGF injections in routine clinical practice and demonstrate that eyes with repeated anti-VEGF treatment for DMO still progress to PDR. Our WCE model suggests that intravitreal anti-VEGF therapy is associated with a reduction in risk of PDR which lasts for 4 weeks after each injection. Baseline DR grade, clinical retinopathy features (IRMA), age, sex, type of diabetes and ethnicity were key prognostic factors. Our findings highlight the risk of PDR progression after intravitreal anti-VEGF therapy has been initiated or stopped and are, together with the CIRs of PDR, of clinical significance for the management and monitoring of DMO patients.

## Supplementary material

10.1136/bmjophth-2025-002234 online supplemental file 1

## Data Availability

No data are available.
