# Supplementary material for: Impact of anti-VEGF treatment for diabetic macular oedema on progression to proliferative diabetic retinopathy: data-driven insights from a multicentre study
Source: BMJ Open Ophthalmol. 2025 Jul 16;10(1):e002234. doi: 10.1136/bmjophth-2025-002234 (PMC12273153; doi:10.1136/bmjophth-2025-002234)
Supplement: online supplemental file 1 [file bmjophth-10-1-s001.pdf]

# Impact of Anti-VEGF Treatment for Diabetic Macular Oedema on Progression to Proliferative Diabetic Retinopathy: Data-driven Insights from a Multicentre Study

## Supplementary Material

*Supplementary methods*

The use of Electronic Health Records (EHR) to assess the effect of diabetic macular oedema (DMO) treatment with intravitreal anti-vascular endothelial growth factor (VEGF) on the risk of development of proliferative diabetic retinopathy (PDR) is challenging due to variation in number and intervals of treatments (exposures) between patients at the point of care. In this contex, conventional models tend to either ignore time varying exposures, or use time-fixed summary measures of exposure (e.g. number of injections at start of follow-up).[1,2] Ignoring exposures or treatments leads to underfitting and potentially biased estimates. And a simple cumulative exposure based on the sum of the number of previous exposures assumes an equal and permanent effect (even after treatment discontinuation) on risk of PDR of all previous exposures. The weighted cumulative exposure (WCE) method assigns weights which reflect the timing of an exposure on the current risk, allowing the assessment of time-dependent exposure patterns by accounting for the cumulative effects of both, exposure numbers and timing. Following the methods from Sylvestre et al.,[1] we modelled the association between exposures (intravitreal anti-VEGF for DMO) and risk of PDR using cubic regression splines modelled in Cox regression. We considered 5 different windows of aetiologically relevant exposure: i) 0.5 years, ii) 1 year, iii) 1.5 years, iv) 2 years, and v) 2.5 years. Models with 1 to 3 interior knots uniformly placed across the time window length were compared for each of the five time windows. The weight function (relative contribution of past exposures on PDR risk) is flexibly estimated from the data, avoids the need of a priori fixed assumptions about exposure-risk relationship, and can fit patterns of delayed effects,

diminishing influence over time, or other complex relationships. For model selection, the model with the best fit assessed by Akaike Information Criterion (AIC) was used for analyses. This data-driven approach avoids potentially misleading assumptions about the precise shape of the exposure-risk relationship and allows for more accurate risk estimation when the etiology is uncertain. The WCE score calculated for each patient represents the weighted sum of all past exposures within the relevant time window, effectively summarising their unique exposure history into a single time-dependent covariate that was then incorporated into the Cox survival model alongside fixed covariates ($WCE_{model}$).

*Supplementary results*

Progression to proliferative diabetic retinopathy by diabetic retinopathy features

In a DR feature-based sub-analysis of treated eyes with severe NPDR only (460/2858, 16%), 71.2% (325/460) of eyes had intraretinal microvascular abnormalities (IRMA), 9.0% (41/460) venous beading, and 20.4% (94/460) 4-quadrant (4Q) dot-blot haemorrhages (DBH). Percentages of PDR development for this group were highest in patients with IRMA and lowest in patients with 4Q DBH (supplementary figure 3). A WCE model (1 knot, 6-month time window exposure) allowing for severe DR features, age, sex, type of diabetes and IMD, only patients with IRMA showed a significant association with a HR of 2.55 (95% CI 1.12-5.79, $p$ = 0.022) for PDR development when compared to 4Q DBH (supplementary table 7). Venous beading features were not associated with PDR development ($p$ = 0.433).

Figure 1. Diagram of exclusions.

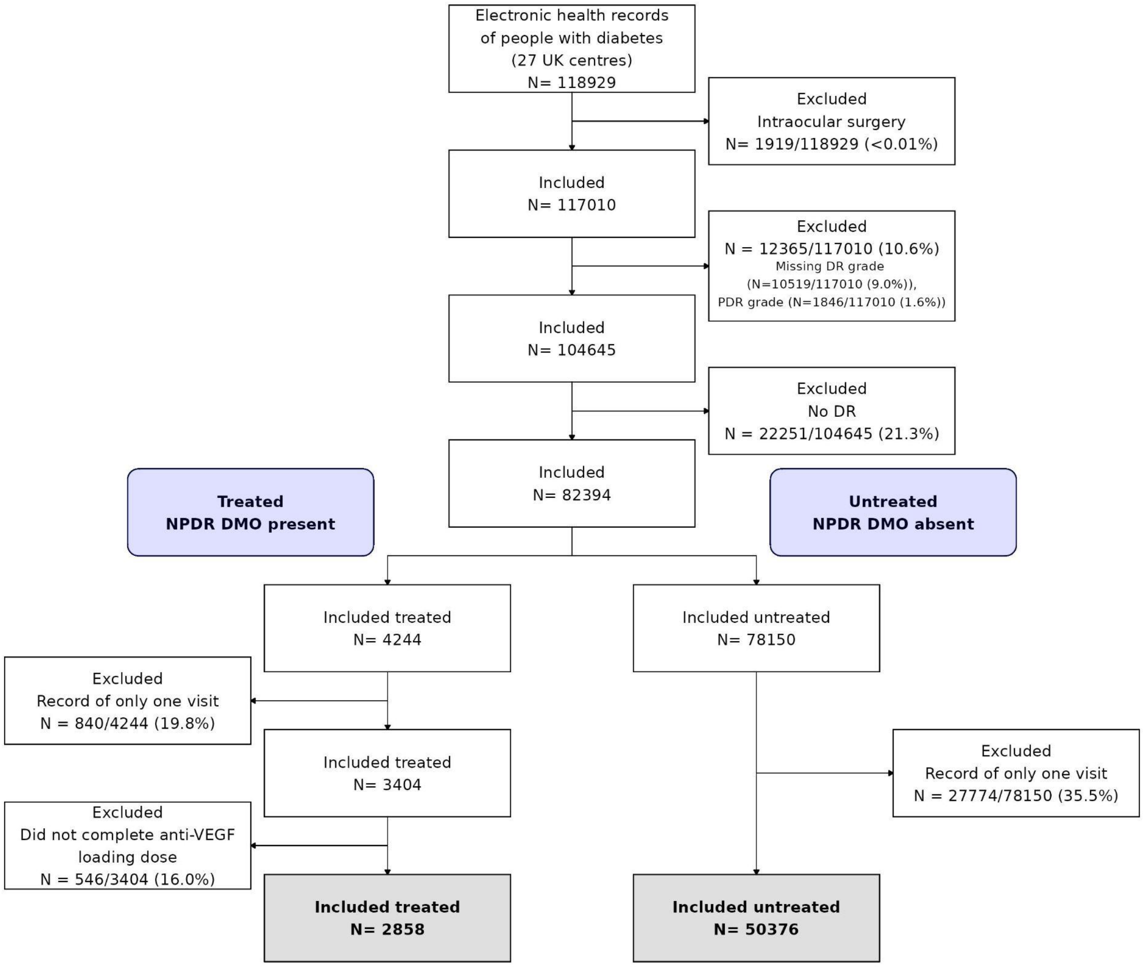

Figure 2. Estimated weight function (solid line) and 95% Confidence Intervals obtained by bootstrapping (grey bands) for the final weighted cumulative exposure model of the association between past intravitreal anti-VEGF exposure and proliferative diabetic retinopathy. The model uses 3 internal knots over a 6-month exposure window. The x-axis is reversed with the origin at week 0 corresponding to time of assessment (red arrow). Hazard ratios associated with specific exposure patterns are derived from the weighted sum of past injections.

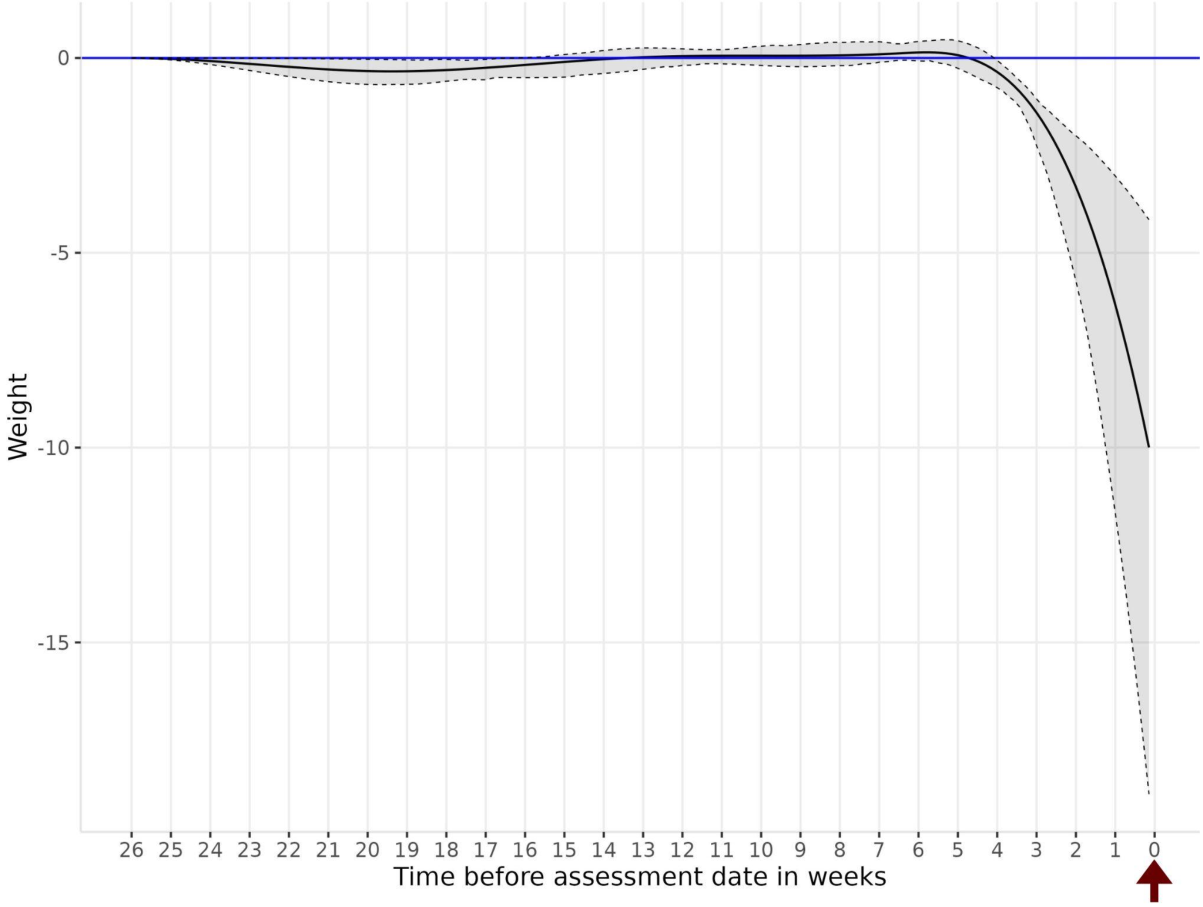

Figure 3. Survival probabilities by diabetic retinopathy features in eyes with severe non-proliferative diabetic retinopathy and diabetic macular oedema treated with intravitreal anti-vascular endothelial growth factor.

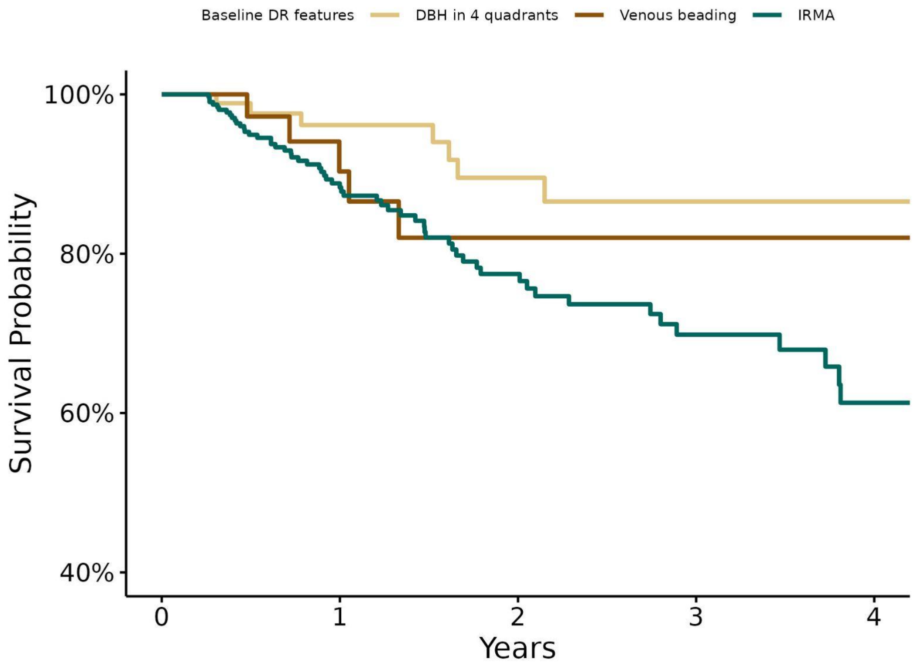


| **N at risk (n events)** | | | | | |
|---|---|---|---|---|---|
| DBH in 4 quadrants | 93 (0) | 56 (3) | 33 (3) | 17 (1) | 8 (0) |
| Venous beading | 41 (0) | 24 (3) | 14 (2) | 9 (0) | 6 (0) |
| IRMA | 325 (0) | 173 (30) | 87 (16) | 50 (7) | 27 (4) |

Table 1. Baseline characteristics with standardised mean differences with 95% confidence intervals.

| Characteristic | Group: Overall N = 53,234 | DMO anti-VEGF-treated N = 2,858 | Treatment-naïve without DMO N = 50,376 | Difference* | 95% CI* |
|---|---|---|---|---|---|
| **Age** | 61 (51, 71) | 64 (57, 72) | 61 (51, 71) | 0.30 | 0.26, 0.34 |
| **Sex** | | | | 0.06 | 0.02, 0.09 |
| Female | 22,145 (42%) | 1,115 (39%) | 21,030 (42%) | | |
| Male | 31,089 (58%) | 1,743 (61%) | 29,346 (58%) | | |
| **Baseline DR grade** | | | | 0.54 | 0.50, 0.58 |
| Mild NPDR | 19,895 (37%) | 463 (16%) | 19,432 (39%) | | |
| Moderate NPDR | 28,931 (54%) | 1,935 (68%) | 26,996 (54%) | | |
| Severe NPDR | 4,408 (8.3%) | 460 (16%) | 3,948 (7.8%) | | |
| **Ethnicity** | | | | 0.22 | 0.18, 0.26 |
| Any other Asian | 1,281 (2.4%) | 61 (2.1%) | 1,220 (2.4%) | | |
| Black | 3,673 (6.9%) | 209 (7.3%) | 3,464 (6.9%) | | |
| Not stated | 14,432 (27%) | 544 (19%) | 13,888 (28%) | | |
| Other | 951 (1.8%) | 71 (2.5%) | 880 (1.7%) | | |
| South Asian | 6,074 (11%) | 313 (11%) | 5,761 (11%) | | |
| White | 26,823 (50%) | 1,660 (58%) | 25,163 (50%) | | |
| **Type of diabetes** | | | | 0.28 | 0.25, 0.32 |
| Type 2 | 34,095 (64%) | 2,129 (74%) | 31,966 (63%) | | |
| Type 1 | 7,078 (13%) | 183 (6.4%) | 6,895 (14%) | | |
| Other | 1,757 (3.3%) | 66 (2.3%) | 1,691 (3.4%) | | |
| Unknown | 10,304 (19%) | 480 (17%) | 9,824 (20%) | | |
| **IMD (quintiles)** | | | | 0.05 | 0.01, 0.09 |
| 1 | 16,545 (31%) | 915 (32%) | 15,630 (31%) | | |
| 2 | 12,906 (24%) | 688 (24%) | 12,218 (24%) | | |
| 3 | 9,121 (17%) | 479 (17%) | 8,642 (17%) | | |
| 4 | 7,804 (15%) | 383 (13%) | 7,421 (15%) | | |
| 5 | 6,858 (13%) | 393 (14%) | 6,465 (13%) | | |

DMO, diabetic macular oedema; VEGF, vascular endothelial growth factor; CI, confidence interval; DR, diabetic retinopathy; NPDR, non-proliferative diabetic retinopathy; IMD, index of multiple deprivation.
Median (IQR) for continuous variables.
Count (column %) for categorical variables.
* Standardised mean difference with 95% confidence intervals.

Table 2. Period incidence rates per 100 person-years for proliferative diabetic retinopathy development by length of follow-up with 95% Confidence Intervals.

| Characteristic | 0-1 yr Non-DMO* | DMO** | 1-2 yr Non-DMO* | DMO** | 2-3 yr Non-DMO* | DMO** | 3-4 yr Non-DMO* | DMO** |
|---|---|---|---|---|---|---|---|---|
| **Overall** | 3.09 (2.96-3.21) | 4.09 (3.48-4.70) | 5.11 (4.93-5.29) | 5.70 (4.75-6.64) | 6.20 (5.97-6.43) | 3.58 (2.57-4.59) | 6.23 (5.96-6.51) | 5.86 (4.09-7.62) |
| **Age group** | | | | | | | | |
| < 55 | 4.12 (3.87- 4.37) | 6.82 (5.02- 8.61) | 6.36 (6.02- 6.71) | 10.09 (7.31-12.87) | 7.86 (7.43- 8.30) | 7.21 (4.10-10.32) | 7.94 (7.44- 8.44) | 9.99 (5.05-14.92) |
| 55 to 65 | 3.24 (2.98- 3.50) | 3.35 (2.37- 4.33) | 5.28 (4.92- 5.64) | 6.33 (4.58- 8.08) | 5.97 (5.52- 6.42) | 3.20 (1.51- 4.90) | 6.24 (5.71- 6.78) | 2.57 (0.54- 4.61) |
| 55 to < 65 | 2.11 (1.95- 2.28) | 3.52 (2.71- 4.32) | 3.85 (3.60- 4.10) | 3.41 (2.34- 4.48) | 4.73 (4.40- 5.07) | 2.23 (1.07- 3.39) | 4.42 (4.03- 4.80) | 6.54 (3.76- 9.31) |
| **Sex** | | | | | | | | |
| Female | 3.11 (2.91-3.30) | 3.36 (2.47-4.25) | 4.82 (4.54-5.09) | 5.27 (3.84-6.70) | 5.77 (5.42-6.12) | 1.83 (0.68-2.98) | 5.98 (5.56-6.40) | 3.98 (1.67-6.30) |
| Male | 3.07 (2.91-3.24) | 4.57 (3.75-5.39) | 5.31 (5.07-5.55) | 5.98 (4.73-7.24) | 6.50 (6.19-6.81) | 4.78 (3.28-6.28) | 6.41 (6.05-6.77) | 7.04 (4.55-9.53) |
| **Baseline DR grade** | | | | | | | | |
| Mild NPDR | 1.17 ( 1.04- 1.30) | 2.13 ( 1.02- 3.23) | 2.05 ( 1.86- 2.24) | 1.92 ( 0.54- 3.31) | 2.94 ( 2.68- 3.21) | 0.00 ( 0.00- 0.00) | 3.65 ( 3.31- 4.00) | 3.70 ( 0.35- 7.05) |
| Moderate NPDR | 3.34 ( 3.16- 3.52) | 3.24 ( 2.58- 3.90) | 5.96 ( 5.70- 6.23) | 5.37 ( 4.25- 6.49) | 6.94 ( 6.61- 7.28) | 3.53 ( 2.31- 4.74) | 6.94 ( 6.54- 7.33) | 6.07 ( 3.86- 8.28) |
| Severe NPDR | 10.88 (10.06-11.69) | 9.65 ( 7.39-11.92) | 14.19 (13.15-15.24) | 11.45 ( 8.16-14.74) | 17.53 (16.19-18.88) | 7.78 ( 3.99-11.57) | 14.61 (13.13-16.09) | 7.10 ( 2.26-11.95) |

DMO; diabetic macular oedema, DR; diabetic retinopathy, NPDR; non-proliferative diabetic retinopathy.
* Shows period incidence rates for patients with no diabetic macular oedema and no anti-VEGF injections.
** Shows period incidence rates for patients with diabetic macular oedema treated at the point of care with anti-VEGF injections.

Table 3. Median (IQR) number of injections during follow-up by baseline diabetic retinopathy (DR) severity.

| | Year 1 | Year 2 | Year 3 | Year 4 | Year 5 |
|---|---|---|---|---|---|
| **Overall** | 6 (4-7) | 5 (3-7) | 5 (3-7) | 5 (3-7) | 5 (3-7) |
| **Baseline DR Severity** | | | | | |
| Mild NPDR | 6 (4-7) | 5 (3-7) | 5 (3-7) | 4 (3-6) | 3 (2-6) |
| Moderate NPDR | 6 (4-7) | 5 (3-6) | 5 (3-6) | 5 (3-7) | 5 (3-7) |
| Severe NPDR | 6 (4-7) | 5 (3-7) | 5 (4-7) | 6 (4-8) | 6 (3-7) |

Table 4. Weighted cumulative exposure models to define best time window and number of knots without making assumptions a priori.

| Model | Time exposure | Knots | AIC | AIC difference* |
|---|---|---|---|---|
| WCE | 0.5 years | 3 | 2,837.6 | 0.0 |
| WCE | 0.5 years | 2 | 2,841.2 | 3.6 |
| WCE | 0.5 years | 1 | 2,842.4 | 4.8 |
| WCE | 1 year | 3 | 2,843.3 | 5.7 |
| WCE | 1 year | 2 | 2,850.9 | 13.3 |
| WCE | 1.5 years | 3 | 2,854.9 | 17.3 |
| WCE | 1 year | 1 | 2,868.2 | 30.6 |
| WCE | 2 years | 3 | 2,869.9 | 32.3 |
| WCE | 1.5 years | 2 | 2,872.2 | 34.6 |
| WCE | 2 years | 2 | 2,883.8 | 46.2 |
| WCE | 1.5 years | 1 | 2,884.0 | 46.4 |
| WCE | 2 years | 1 | 2,891.0 | 53.4 |

AIC; Akaike information criterion.

*AIC of model, minus AIC of best model (minimum AIC)

Table 5. Goodness of fit across modelling strategies.

| Model | Exposure assumptions | AIC | AIC difference* |
|---|---|---|---|
| WCE | Weighted cumulative anti-VEGF injection exposure | 2,837.6 | 0.0 |
| Cox time-dependent | Unweighted cumulative sum of intravitreal anti-VEGF injections (assumes permanent effect) | 2,900.9 | 63.3 |
| Cox | Ignores intravitreal anti-VEGF injections | 2,931.6 | 94.0 |

AIC; Akaike information criterion, WCE; weighted cumulative exposure, VEGF; vascular endothelial growth factor.

*AIC of model, minus AIC of best model (minimum AIC)

Table 6. Mutually adjusted hazard ratios for Cox models. $Cox_{model}$ ignores intravitreal anti-vascular endothelial growth factor injections. $Cox_{tdc}$ introduces intravitreal injections as a time-dependent unweighted cumulative sum of exposures.

| | $Cox_{model}$ | | $Cox_{tdc}$ | |
|---|---|---|---|---|
| **Characteristic** | **HR (95% CI)*** | **p-value** | **HR (95% CI)*** | **p-value** |
| **Baseline DR grade** | | | | |
| Mild NPDR | 1.00 | | 1.00 | |
| Moderate NPDR | 1.92 (1.10, 3.34) | **0.022** | 2.07 (1.19, 3.61) | **0.011** |
| Severe NPDR | 4.33 (2.41, 7.77) | **8.9e-07** | 4.77 (2.66, 8.57) | **1.7e-07** |
| **Age (per 5 years)** | 0.90 (0.85, 0.96) | **0.001** | 0.91 (0.85, 0.96) | **0.001** |
| **Sex** | | | | |
| Female | 1.00 | | 1.00 | |
| Male | 1.34 (1.00, 1.81) | 0.052 | 1.36 (1.00, 1.83) | **0.047** |
| **Type of diabetes** | | | | |
| Type 2 | 1.00 | | 1.00 | |
| Type 1 | 2.04 (1.34, 3.12) | **9.8e-04** | 2.05 (1.34, 3.13) | **8.9e-04** |
| Other | 1.30 (0.53, 3.18) | 0.567 | 1.25 (0.51, 3.05) | 0.632 |
| Unknown | 0.93 (0.63, 1.38) | 0.730 | 0.89 (0.60, 1.31) | 0.544 |
| **Ethnicity** | | | | |
| White | 1.00 | | 1.00 | |
| South Asian | 1.00 (0.64, 1.57) | 0.998 | 0.89 (0.57, 1.40) | 0.617 |
| Black | 0.48 (0.22, 1.04) | 0.063 | 0.44 (0.20, 0.95) | **0.036** |
| Any other Asian | 0.81 (0.30, 2.20) | 0.677 | 0.83 (0.30, 2.25) | 0.710 |
| Other | 0.34 (0.08, 1.38) | 0.132 | 0.33 (0.08, 1.36) | 0.125 |
| Not stated | 1.00 (0.70, 1.44) | 0.996 | 1.02 (0.71, 1.47) | 0.902 |
| **IMD (quintiles)** | | | | |
| 1 | 1.00 | | 1.00 | |
| 2 | 1.03 (0.72, 1.47) | 0.869 | 1.06 (0.74, 1.52) | 0.756 |
| 3 | 1.0 (0.66, 1.50) | 0.979 | 1.05 (0.70, 1.59) | 0.800 |
| 4 | 0.86 (0.56, 1.33) | 0.512 | 0.91 (0.59, 1.40) | 0.665 |
| 5 | 0.62 (0.37, 1.02) | 0.059 | 0.64 (0.39, 1.07) | 0.088 |
| **Intravitreal injection** | † | | 0.89 (0.85, 0.93) | **3.6e-08** |

*HR; hazard ratio, CI; confidence interval

†Ignores anti-vascular endothelial growth factor injections

Table 7. Mutually adjusted hazard ratios in treated eyes with severe non-proliferative diabetic retinopathy (NPDR) including diabetic retinopathy features at baseline.

| Characteristic | HR (95% CI)* | p-value |
|---|---|---|
| **Severe DR features** | | |
| DBH in 4 quadrants | 1.00 | |
| IRMA | 2.55 (1.12, 5.79) | **0.022** |
| Venous beading | 1.56 (0.50, 4.88) | 0.433 |
| **Age (per 5-year rise)** | 0.88 (0.79, 0.99) | **0.029** |
| **Sex** | | |
| Female | 1.00 | |
| Male | 1.51 (0.89, 2.54) | 0.117 |
| **Type of diabetes** | | |
| Type 2 | 1.00 | |
| Type 1 | 1.13 (0.50, 2.54) | 0.767 |
| Other | 1.54 (0.35, 6.70) | 0.561 |
| Unknown | 0.79 (0.39, 1.60) | 0.498 |
| **IMD quintiles** | | |
| 1 (most deprived) | 1.00 | |
| 2 | 1.23 (0.65, 2.34) | 0.516 |
| 3 | 1.30 (0.62, 2.71) | 0.480 |
| 4 | 0.81 (0.38, 1.75) | 0.584 |
| 5 (least deprived) | 0.74 (0.32, 1.71) | 0.473 |

*HR; hazard ratio, CI; confidence interval

DR; diabetic retinopathy, DBH; dot-blot haemorrhages, IRMA; intraretinal microvascular abnormalities, IMD; index of multiple deprivation.

## REFERENCES


1. Sylvestre, M.-P. & Abrahamowicz, M. Flexible modeling of the cumulative effects of time-dependent exposures on the hazard. *Stat Med* **28**, 3437–3453 (2009).
2. Kelly, T.-L., Salter, A. & Pratt, N. L. The weighted cumulative exposure method and its application to pharmacoepidemiology: A narrative review. *Pharmacoepidemiol Drug Saf* **33**, e5701 (2024).
